# Supplementary material for: Molars to Medicine: A Focused Review on the Pre-Clinical Investigation and Treatment of Secondary Degeneration following Spinal Cord Injury Using Dental Stem Cells
Source: Cells. 2024 May 10;13(10):817. doi: 10.3390/cells13100817 (PMC11119219; doi:10.3390/cells13100817)
Supplement: Supplementary file 1 [file cells-13-00817-s001.zip › Supplementary Table S1.pdf]

Supplementary Table S1. English search strategies for focused review.

**PubMed 29.02.2024**	
**Search strategies**	**Results**
#1: "dental pulp stem cell*"[Title/Abstract] OR "stem cell from human exfoliated deciduous teeth"[Title/Abstract] OR "human exfoliated deciduous teeth"[Title/Abstract] OR "exfoliated deciduous teeth"[Title/Abstract] OR "dental stem cell*"[Title/Abstract] OR "dental pulp cell*"[Title/Abstract] OR "dental pulp stem cell*"[Title/Abstract] OR "dental pulp stem cell*"[Title/Abstract] OR "human dental pulp stem cell*"[Title/Abstract] OR "human dental pulp"[Title/Abstract] OR "DPSC*"[Title/Abstract] OR "HDPC*"[Title/Abstract] OR "dental follicle stem cell*"[Title/Abstract] OR "dental follicle progenitor cell*"[Title/Abstract] OR "DFPC*"[Title/Abstract] OR "DFSC*"[Title/Abstract] OR "stem cells from apical papilla"[Title/Abstract] OR "stem cell from apical papilla"[Title/Abstract] OR "SCAP"[Title/Abstract] OR "periodontal ligament stem cell*"[Title/Abstract] OR "PDLSC*"[Title/Abstract]	8,676
#2: "spinal cord injury"[Title/Abstract] OR "spinal injury"[Title/Abstract] OR "spinal cord trauma"[Title/Abstract] OR "spinal cord transection"[Title/Abstract] OR "spinal cord laceration"[Title/Abstract] OR "post traumatic myelopathy"[Title/Abstract] OR "spinal cord contusion"[Title/Abstract] OR "spinal cord hemi-contusion"[Title/Abstract] OR "spinal cord hemi-section"[Title/Abstract]	49,092
**#3**: #1 AND #2	62
**#4**: #3 NOT Review [sb]	50
